# Trimetazidine and exercise provide comparable improvements to high fat diet-induced muscle dysfunction through enhancement of mitochondrial quality control

**DOI:** 10.1038/s41598-021-98771-6

**Published:** 2021-09-27

**Authors:** Wenliang Zhang, Baiyang You, Dake Qi, Ling Qiu, Jeffrey W. Ripley-Gonzalez, Fan Zheng, Siqian Fu, Cui Li, Yaoshan Dun, Suixin Liu

**Affiliations:** 1 Division of Cardiac Rehabilitation, Department of Physical Medicine and Rehabilitation, Xiangya Hospital of Central South University, 87 Xiangya Road, Changsha, Hunan 410008 People’s Republic of China; 2 College of Pharmacy, University of Manitoba, Winnipeg, Canada; 3 Division of Preventive Cardiology, Department of Cardiovascular Medicine, Mayo Clinic, Rochester, MN USA; 4 National Clinical Research Center for Geriatric Disorders, Xiangya Hospital of Central South University, Changsha, Hunan People’s Republic of China

**Keywords:** Endocrinology, Drug development, Preclinical research

## Abstract

Obesity induces skeletal muscle dysfunction. The pathogenesis of which appears to substantially involve mitochondrial dysfunction, arising from impaired quality control. Exercise is a major therapeutic strategy against muscle dysfunction. Trimetazidine, a partial inhibitor of lipid oxidation, has been proposed as a metabolic modulator for several cardiovascular pathologies. However, the effects of Trimetazidine on regulating skeletal muscle function are largely unknown. Our present study used cell culture and obese mice models to test a novel hypothesis that Trimetazidine could improve muscle atrophy with similar results to exercise. In C2C12 cells, high palmitic acid-induced atrophy and mitochondrial dysfunction, which could be reversed by the treatment of Trimetazidine. In our animal models, with high-fat diet-induced obesity associated with skeletal muscle atrophy, Trimetazidine prevented muscle dysfunction, corrected metabolic abnormalities, and improved mitochondrial quality control and mitochondrial functions similarly to exercise. Thus, our study suggests that Trimetazidine successfully mimics exercise to enhance mitochondrial quality control leading to improved high-fat diet-induced muscle dysfunction.

## Introduction

Muscle atrophy^1^ is characterized by a loss of muscle mass and strength leading to functional limitation and increased physical disability^2,3^. In obesity, mitochondrial dysfunction and damage due to excessive fatty acid infiltration and accumulation in skeletal muscle may contribute to muscle atrophy^4–6^. Mitochondrial dysfunction triggers catabolic signaling pathways to induce and/or exacerbate muscle atrophy^7,8^. Mitochondrial integrity relies on the efficiency of mitochondrial quality control (MQC) processes, and their morphology is regulated by continuous fusion, fission, and mitophagy^9–13^. Thus, mitochondrial quality control is an important parameter in evaluating muscle atrophy during obesity. Exercise effectively improves muscle mass, strength, and physical performance^14^. It could decrease transcription of atrogenes, such as Muscle Atrophy F-boX protein (Atrogin1) and Muscle RING-Finger Protein-1(MuRF1). Our previous study also indicated that exercise remodels MQC and improves mitochondrial functions^15,16^.

Trimetazidine (TMZ) is an anti-angina medication. It inhibits FFA oxidation and promotes glucose oxidation by selectively acting on the mitochondrial long-chain 3-ketoacyl-CoA thiolase. TMZ also inhibits oxidative stress to preserve mitochondrial structure and their functions^17^. Thus, in the heart, TMZ treatment has a protective effect on preventing ischemic injury^18^. TMZ also has a beneficial effect on skeletal muscle. The treatment of TMZ significantly increases the strength of skeletal muscle in aged animals^19^. It also enhances the fast-to-slow myofibre phenotype shift, peroxisome proliferator-activated receptor-gamma coactivator 1-α (PGC1-α) upregulation, oxidative metabolism, and mitochondrial biogenesis in C26-bearing mice^20^. TMZ upregulated the AMP-activated protein kinase (AMPK)/PGC1α signalling pathway to promote mitochondrial biogenesis and activate autophagy in cachectic tumor-bearing mouse skeletal muscle^21^. Further research provided evidence that TMZ may also stimulate signaling in reducing protein degradation and increasing MyHC mRNA expression^22^.

Our previous research indicated that TMZ alone or with exercise improves muscle function and the efficiency of mitochondrial quality control processes^16^. Our present study aims to further investigate whether exercise and TMZ respectively improve muscle dysfunction induced by a high-fat diet (HFD). We would focus on studying the effects of TMZ on regulating MQC in both in vitro and in vivo atrophy models. We hypothesize that TMZ could be a paralleled therapy with exercise for muscle dysfunction.

## Results

### TMZ prevents palmitate (PA)-induced muscle cell damage

**Figure 1:**
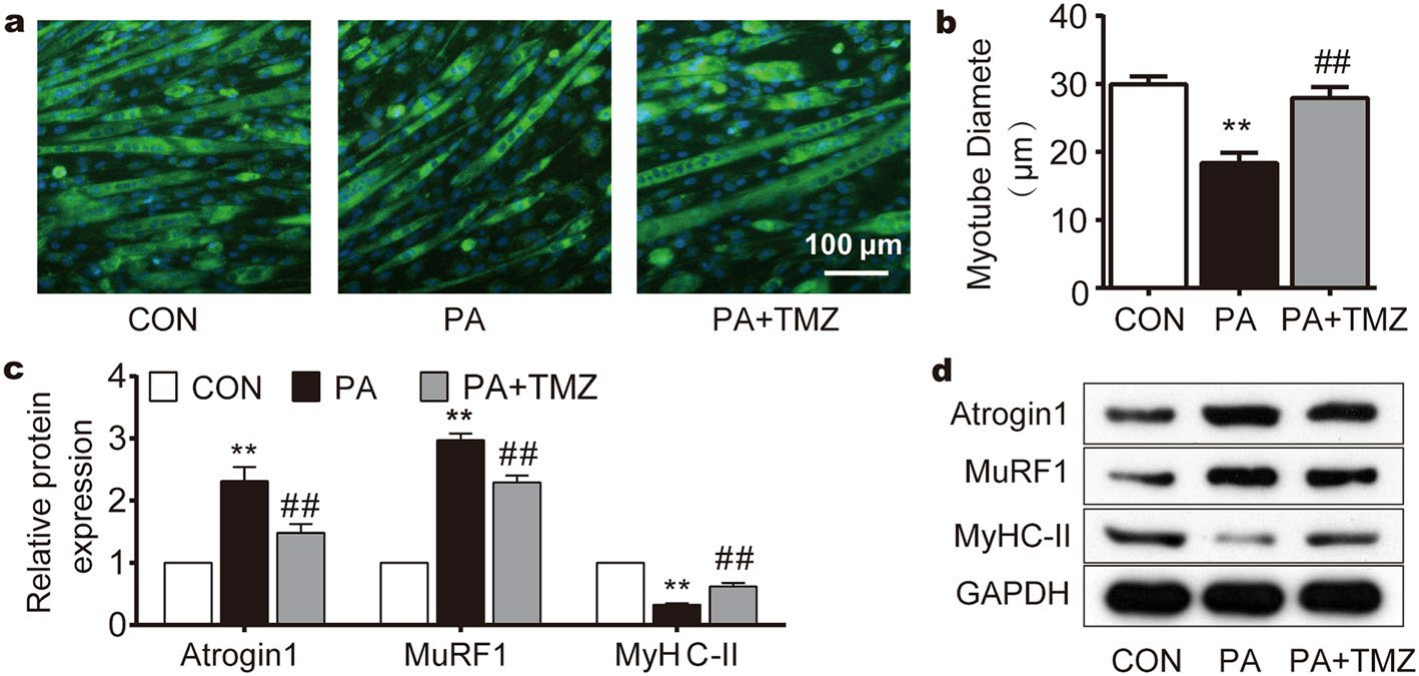
TMZ prevents palmitate (PA)-induced muscle cell damage. *Con* control group without palmitate treatment, *PA* skeletal muscle cells treated with palmitate, *TMZ* skeletal muscle cells treated with trimetazidine. (**a**, **b**) MyHC-II immunofluorescence staining was performed to observe the myotube cell morphology and myotube diameter with a 100 μm scale. (**c**, **d**) MyHC-II, MuRF1, and Atrogin1 protein expression were detected by Western blot. *, ** represented p < 0.05, p < 0.01 in comparison with the Con group, respectively. ^#^, ^##^ represented p < 0.05, p < 0.01 in comparison with the PA  group, respectively.

Myosin heavy chain II (MyHC-II) was stained to detect myotube cytoarchitecture. Following high PA treatment, myotubes were sparsely distributed and their diameters became smaller compared to the control group (Fig. 1a). However, TMZ treatment resisted the effects of PA and successfully rescued the cytoskeletal derangement (Fig. 1a,b). High PA also reduced the protein expression of MyHC-II while increasing the muscle atrophy factors, such as MuRF1 and Atrogin1 (Fig. 1c,d). The concurrent TMZ treatment with high PA partially reversed these changes in MyHC-II, MuRF1, and Atrogin1 (all p < 0.01), suggesting that TMZ could reverse PA-induced muscle damage in vitro.

### TMZ promotes PA-attenuated MQC and functions in muscle cells

**Figure 2:**
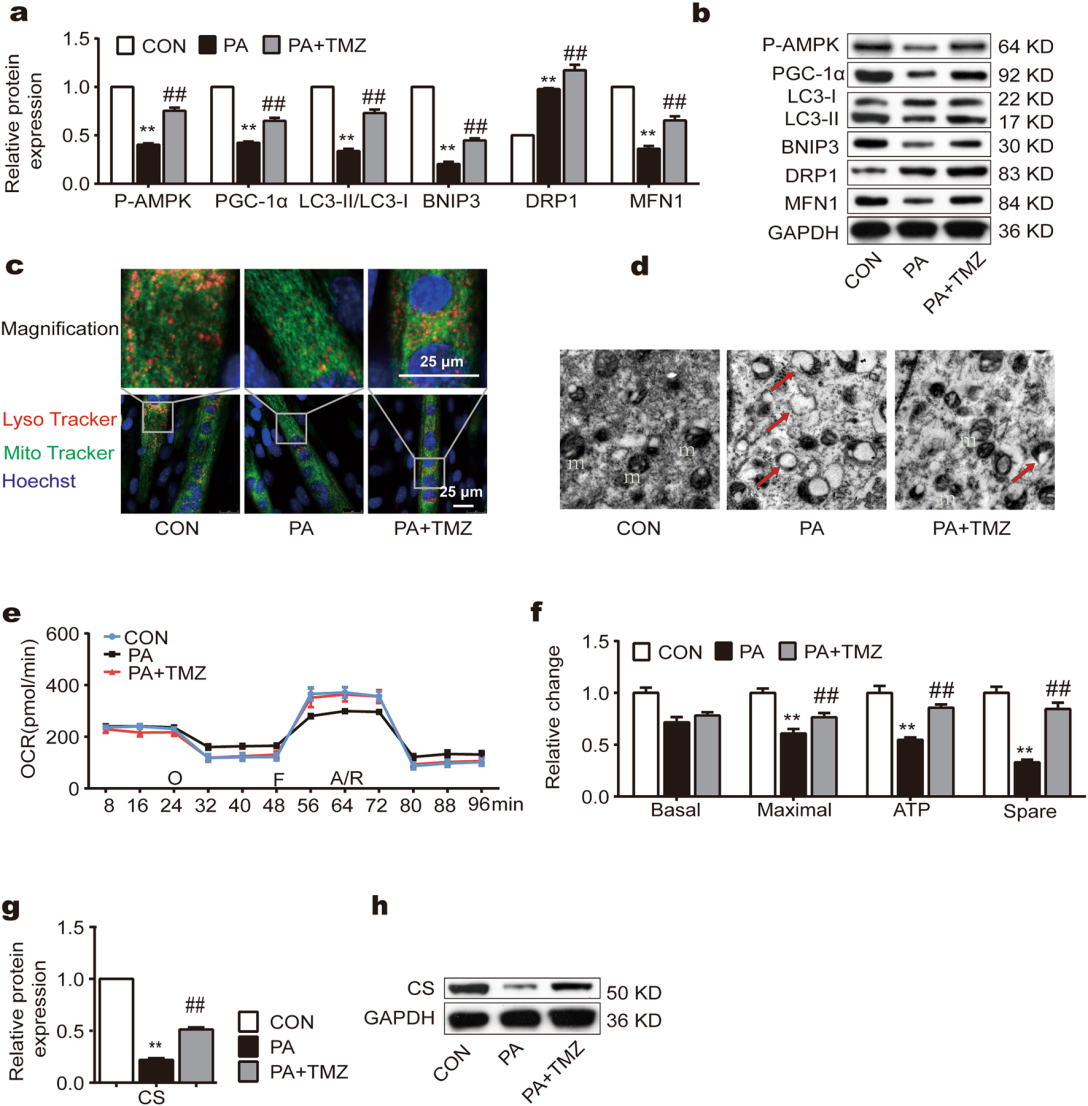
TMZ promotes PA-attenuated mitochondrial quality control (MQC) and functions in muscle cells. (**a**, **b**) P-AMPK, PGC-1α, LC3II/LC3I, BNIP3, MFN1, and DRP1 protein expression were determined by Western blot. (**c**) Laser scanning confocal microscope by using LysoTracker and mitoTacker. The colocalizations of lysoTracker and mitoTracker represented mitolysosomes (scale bar = 25 μm). (**d**) Transmission electron microscope imaging. The damaged mitochondria were indicated in TEM images (red arrow). (**e**, **f**) OCR: cellular oxygen consumption. m: mitochondria. (**g**, **h**) CS protein expression was determined by Western blot. *, ** represented p < 0.05, p < 0.01 in comparison with the Con group, respectively; ^#^, ^##^ represented p < 0.05, p < 0.01 in comparison with the PA group, respectively.

PA significantly affected mitochondrial biogenesis, mitophagy, and fission/fusion characterized with reduced protein levels in Phosphorylated-adenosine 5-monophosphate activated protein kinase (P-AMPK), PGC-1α, microtubule-associated protein 1 light chain 3—II and I (LC3-II/LC3-I), BCL2/adenovirus E1B interacting protein 3 (BNIP3), mitofusin-1 (MFN1), and an increased level in dynamin-related protein 1 (DRP1) (Fig. 2a,b). However, the concurrent treatment of TMZ with PA resisted these alterations and improved MQC (all p < 0.01, Fig. 2a,b). By using LysoTraker and MitoTracker staining, we also found that mitolysosomes following PA treatment were less compared to control and they were more abundant in the PA + TMZ group (Fig. 2c). The transmission electron microscopy (TEM) images further indicated that PA treatment caused mitochondrial damage, characterized by edema and disappeared crista, which was reversed by TMZ (Fig. 2d).

Oxygen consumption rate (OCR) is a key marker for mitochondrial function. Here, we also investigated OCR in muscle cells following PA treatment with or without TMZ. We observed that although there was no difference in the basal OCR between the PA and PA + TMZ groups, the maximal OCR, the spare OCR, and the adenosine triphosphate (ATP) production were all increased in the PA + TMZ group (all p < 0.01, Fig. 2e,f). Further shown in Fig. 2g,h, PA suppressed the expressions of citrate synthase (CS) level in the skeletal muscle cells but the concurrent TMZ treatment inhibited these aberrant changes induced by PA (all p < 0.01).

### TMZ prevents metabolic dysfunction and muscle atrophy in mice following high-fat diet feeding similarly to exercise

**Figure 3:**
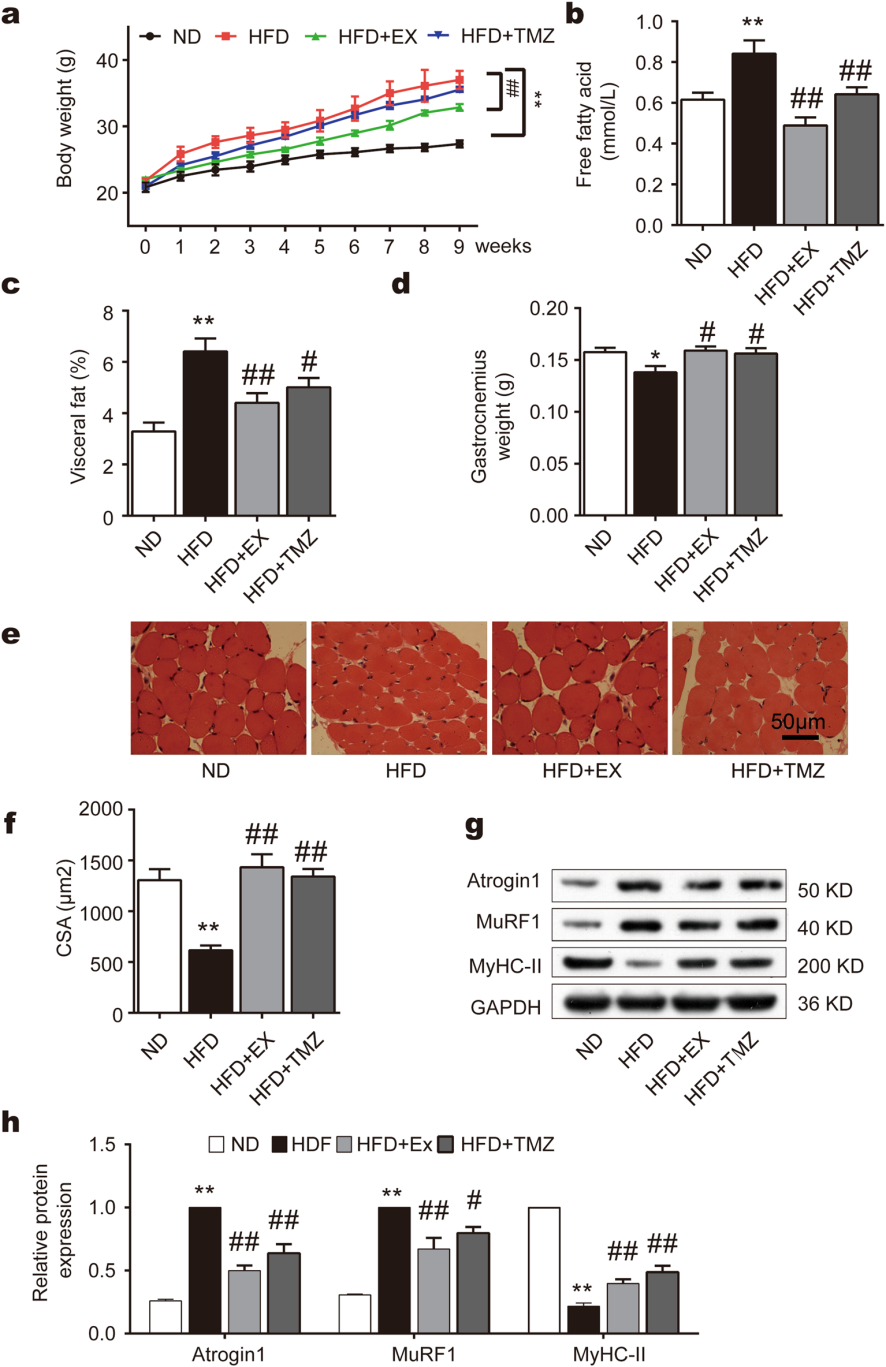
TMZ prevents metabolic dysfunction and muscle atrophy in mice following high-fat diet feeding similarly to exercise. (**a**) Body weights of normal diet-group (ND), high-fat diet-group (HFD), high-fat diet + exercise-group (HFD + Ex), and high-fat diet + trimetazidine-group (HFD + TMZ) during eight weeks of intervention. (**b**) The concentration of free fatty acids (FFA) in fasting plasma. (**c**) Visceral fat was dissected and weighed. (**d**) Gastrocnemius muscle was dissected and weighed. (**e**, **f**) Hematein and eosin (HE) staining of skeletal muscle tissues. Muscle fiber cross-sectional area was calculated by the image analysis software. (**g**, **h**) MyHC-II, MuRF1, and Atrogin1 protein expression were determined by Western blot. *, ** represented p < 0.05, p < 0.01 in comparison with ND group, respectively; ^#^, ^##^ represented p < 0.05, p < 0.01 in comparison with HFD group, respectively.

HFD for 8 weeks induced obesity in mice, which was significantly reversed by exercise or TMZ (Fig. 3a). However, TMZ did not affect body weight gain as exercise (Fig. 3a). Interestingly, both exercise and TMZ downregulated plasma fatty acid levels and visceral fat accumulation following high-fat diet feeding (Fig. 3b,c), suggesting that TMZ may have a similar effect as exercise on preventing the occurrence of metabolic dysfunction. Gastrocnemius muscle weight was reduced following the HFD feeding which was fully reversed by either exercise or TMZ treatment (Fig. 3d). Hematein-eosin (HE) staining images exhibited a cross-sectional area of skeletal muscle fibers in Fig. 3e. The area of muscle fiber was significantly reduced following high-fat diet feeding and the reduction was fully reversed by either exercise or TMZ treatment (all p < 0.01, Fig. 3f). Accompanied with these morphological changes, a high-fat diet also upregulated the expression of muscle atrophy factors, such as MuRF1 and Atrogin1 while reduced the expression of MyHC-II in vivo (Fig. 3g,h). These alterations were partially reversed by TMZ and exercise (Fig. 3g,h), suggesting that TMZ resists muscle atrophy induced by a high-fat diet similar to exercise in in vivo animal models.

### TMZ regulates MQC and improves mitochondrial functions in HFD mice

**Figure 4:**
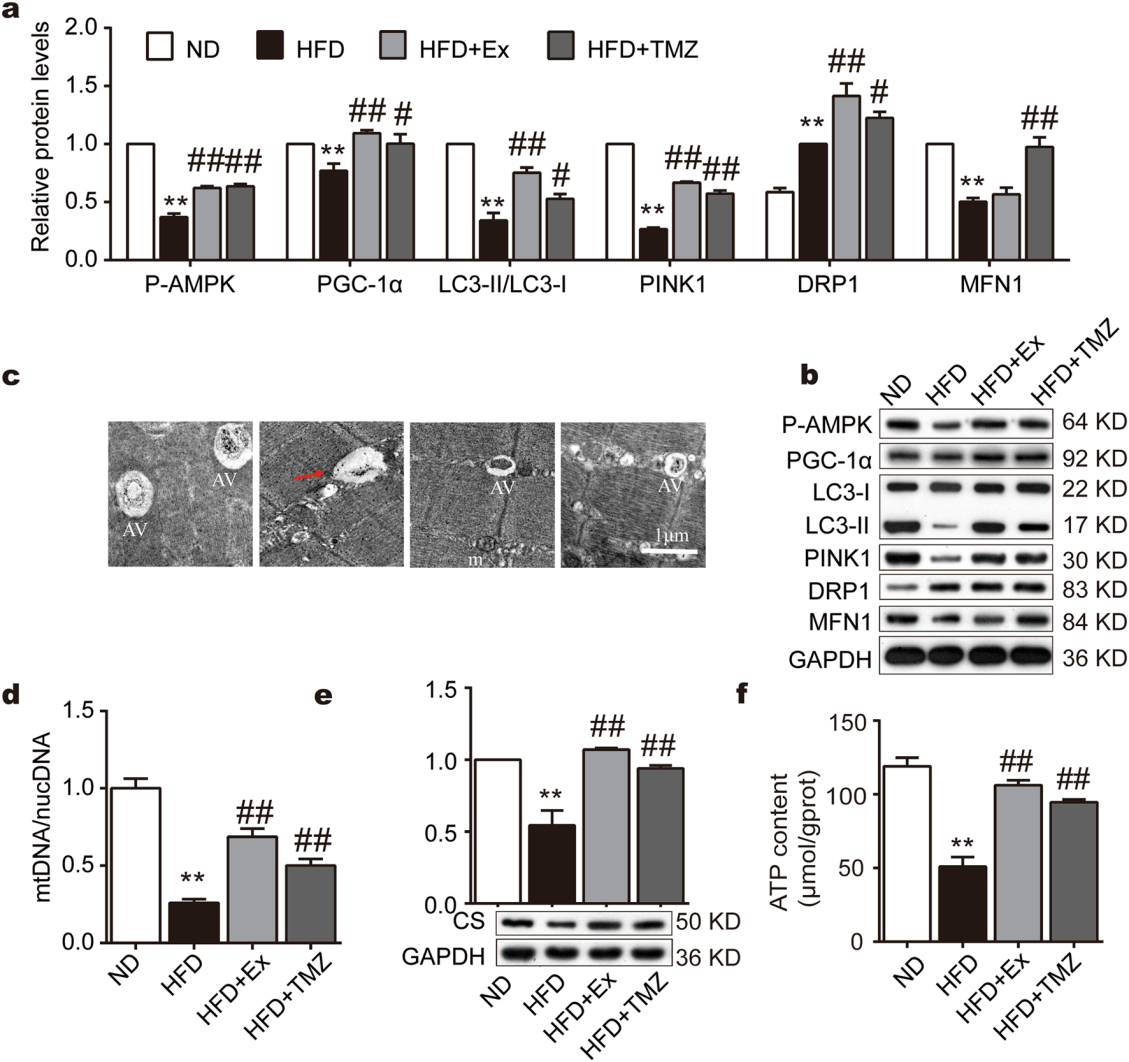
Comparative to exercise, TMZ regulates MQC and improves mitochondrial functions in HFD mice. (**a**, **b**) P-AMPK, PGC-1α, LC3II/LC3I, PINK1, MFN1, and DRP1 protein expressions were determined by Western blot. (**c**) Transmission electron microscope observation of mouse skeletal muscle tissue with 1 μm scale. *AV* autophagic vacuole, *m* mitochondria. (**d**) Mitochondrial DNA/nuclear DNA was determined by PCR. € CS protein expression was determined by Western blot. (**f**) ATP content were measured. *, ** represented p < 0.05, p < 0.01 in comparison with ND group, respectively; ^#^, ^##^ represented p < 0.05, p < 0.01 in comparison with HFD group, respectively.

We next investigated whether TMZ regulates oxidative stress via mediating mitochondrial quality and functions. HFD caused a reduction in the expression of P-AMPK, PGC-1α, LC3II/LC3I, PTEN induced kinase 1 (PINK1), and MFN1. It also suppressed mitochondrial DNA content, CS, and ATP levels. Exercise or TMZ successfully resisted these changes (all p < 0.01, Fig. 4a,b,d–f).

By using transmission electron microscopy, we also observed an abnormal morphological change induced by HFD. The NC group had abundant autophagic vacuoles which were rarely found in the HFD group. In addition, HFD induced mitochondrial edema and crista disappearance. After exercise or TMZ treatment, autophagic vacuoles remained and mitochondrial damage was reduced (Fig. 4c).

### TMZ regulates muscle function in the HFD mice similarly to exercise

**Figure 5:**
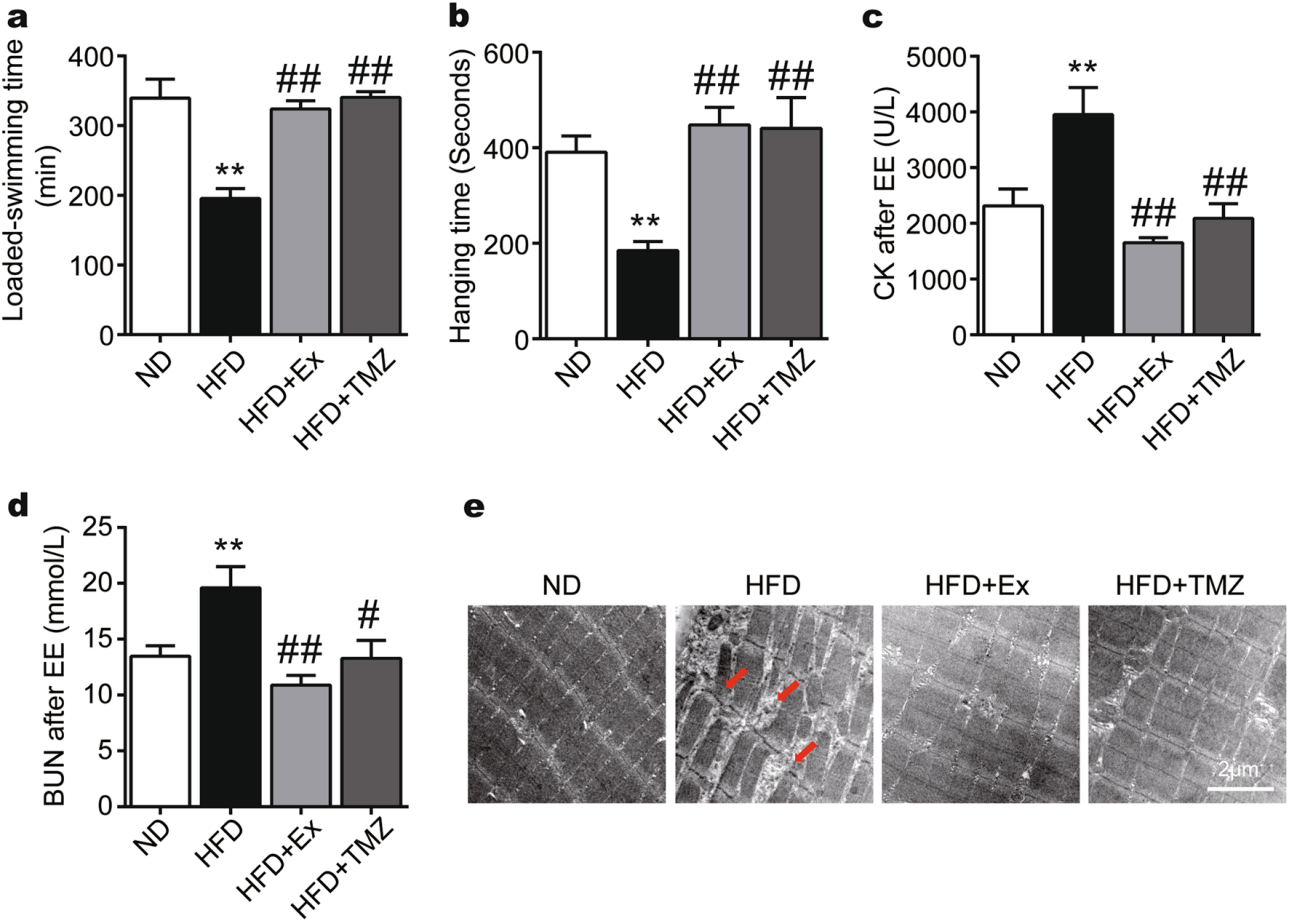
TMZ regulates muscle function in the HFD mice comparably to exercise. (**a**) The forced weight-loaded swimming time was recorded as exercise capacity (*EE* exhaustive exercise). (**b**) The limb strength of the skeletal muscles was tested by the inverted screen test, meanwhile the fall-off time was recorded. (**c**, **d**) Plasma creatine kinase (CK) concentrations and plasma urea nitrogen (BUN) were observed after EE. (**e**) The skeletal structures of HFD mice were observed by transmission electron micrograph after EE with a scale of 2 μm. The red arrow represented the disordered and ruptured arrangement of the skeletal muscle fibers. *, ** represented p < 0.05, p < 0.01 in comparison with ND group, respectively; ^#^, ^##^ represented p < 0.05, p < 0.01 in comparison with HFD group, respectively.

TMZ treatment improved skeletal muscle function in HFD mice with similar effects to exercise (Fig. 5). HFD reduced the hanging time of the forced weight-loaded swimming (Fig. 5a). It also attenuated the time inverted screen test compared to the ND group (Fig. 5b). The functional deficiency of skeletal muscle was associated with augmented creatine kinase (CK) and blood urea nitrogen (BUN) release (Fig. 5c,d). TMZ treatment and exercise both reversed skeletal muscle dysfunction and CK and BUN levels, suggesting that TMZ may have comparable effects to exercise against muscle damage and dysfunction induced by a high-fat diet (all p < 0.01, Fig. 5c,d). Electron microscope images also indicated that HFD caused muscle fiber damage in isolated skeletal muscle from HFD mice which was reversed by either exercise or TMZ treatment (Fig. 5e).

## Discussion

Obesity is often associated with skeletal muscle dysfunction but the mechanisms are largely unknown. Our present study indicated that the anti-angina medication, TMZ could improve high palmitate-induced cell atrophy in C2C12 myotubes via enhancing mitochondrial functions as well as MQC. In in vivo mouse models, TMZ also resisted the development of muscle atrophy and dysfunction induced by HFD comparable to exercise, suggesting that TMZ might be a potential therapy in the treatment of muscle atrophy in addition to exercise.

Muscle atrophy is associated with aging, obesity, diabetes, and cancer^3^. Many factors, such as cellular senescence, oxidative stress, mitochondrial dysfunction, fat accumulation, low-grade inflammation, inadequate nutrition, hormonal changes, etc., may contribute to the development of muscle dysfunction^23–28^. However, muscle wasting is mainly caused by excessive catabolism of myofibrillar proteins. It principally depends on: (a) the ubiquitin–proteasome system; and (b) the autophagy–lysosomal system. The E3 ubiquitin ligases Atrogin-1 and MuRF1 are two key players to promote the degradation of proteins and contribute to skeletal muscle dysfunction. In aminal models, metabolic abnormalities induced by saturated fatty acid were accompanied by muscle dysfunction in mice^5,8,29^. Our present study revealed that high palmitic acid or a high caloric diet rich in palmitic acid promoted muscle dysfunction characterized by a reduction in muscle mass and muscle fiber size. These pathological muscle changes were related to an upregulation in atrophic factors (i.e. Atrogin-1/MAFbx and MuRF1) and proteolysis^29,30^.

Mitochondrial dysfunction arising from impaired quality control is critically involved in the pathogenesis of muscle dysfunction^4,31^. Derangements at any level of the MQC axis could result in mitochondrial dysfunction, energy shortage, and ultimately the loss of cell viability. MQC including mitochondrial biogenesis, dynamics, and autophagy cooperate to ensure mitochondrial homeostasis^9,10^. Mitochondrial dynamics control mitochondrial shape and functions by coordinating the opposing processes of mitochondrial fusion and fission^32^. Several studies have provided evidence that failure in mitochondrial dynamics leads to a negative consequence for the maintenance of muscle mass and function^33–36^. Autophagy activation is crucial to avoid an accumulation of dysfunctional organelles, unfolded, and toxic proteins that lead to muscle dysfunction. Moreover, lipotoxicity, which may result from a HFD, contributes to mitochondrial dysfunction and ROS production. Palmitate induced delayed myoblast differentiation, insulin resistance, cellular senescence, impaired autophagic flux, and increased Atrogin-1 and MuRF1 gene expression, which provide a suitable sarcopenia model^37–39^. Here, we showed that PA and HFD significantly altered mitochondrial integrity and attenuated mitochondrial turnover due to insufficient mitochondrial biogenesis, fission/fusion, and/or defective autophagic removal, characterized with reduced protein levels in P-AMPK, PGC-1α, LC3-II/LC3-I, BNIP3, MFN1. HFD and PA increased levels of DRP1, a marker of stimulated mitochondrial fission. This increase in DRP1 suggests that a HFD could trigger mitochondrial fission in skeletal muscles. These findings support previous research^40,41^ in that mitochondrial fission is stimulated via the upregulation of DRP1 expression in mouse gastrocnemius muscle and C2C12 mouse myoblasts. The HFD group had lower mitochondrial function. Since the mitochondrial quality has been deteriorated after a HFD, the ATP content, may in turn be reduced. This supports previous studies^7^, where HFD mice experience a low level of p-AMPK along with low Citrate Synthase, mitochondrial respiratory complexes I and II activity which may indicate lower ATP production. Since mitochondrial dysfunction contributes to the loss of muscle mass and muscle dysfunction, MQC is a potential target in the development of novel therapeutic interventions against muscle dysfunction.

Both clinical and experimental studies have demonstrated that exercise is an efficient strategy against muscle dysfunction induced by metabolic dysfunction. Exercise not only corrects energy metabolism but also increases muscle mass, strength, and physical performance^14,42–44^. We confirmed that exercise resists muscle dysfunction and dysfunction following metabolic abnormalities. Exercise also improved physical performance, i.e., the hanging time and forced weight-loaded swimming time of the obese mice. Previous studies suggested that exercise may increase mitochondrial content and improve mitochondrial functions through regulating mitochondrial fusion, fission, and autophagy. We previously also found that exercise enhances MQC and improves exercise capacity in healthy C57BL/6 mice^15,16^. In the present study, exercise resists the impairment of MQC in HFD mice via enhancing MQC-related parameters P-AMPK, PGC-1α, LC3II/LC3I, PINK1, and MFN1, suggesting that exercise successfully prevents metabolic dysfunction-induced morphological and functional deficiency in muscle cells. Although exercise is an efficient strategy against muscle dysfunction, it is not applicable for all conditions. The patients who are bedridden or immobilized, hospitalized due to orthopaedic surgery, disabled or aging^45–47^ are normally not well suited for exercise. Thus, a new therapeutic strategy to replace or parallel with exercise would be important.

TMZ has been widely used in patients with coronary artery disease. TMZ also prevented C2C12 myotubes from TNF-α or starvation-induced damage^22^ and enhanced the fast-to-slow myofibre phenotype shift in C26-bearing mice^20^. Our present study found that TMZ reversed PA-induced muscle cell damage and HFD-induced muscle dysfunction by upregulating MyHC-II while downregulating atrophic factors. More importantly, TMZ resisted the development of muscle atrophy and dysfunction in HFD mice by improving mitochondrial functions. Previous studies also indicated that TMZ facilitated myoblast differentiation and myogenesis through upregulation of the AMP-activated protein kinase (AMPK)/PGC1α signaling pathway which may promote mitochondrial biogenesis in aging mice^48^. Thus, the effects of TMZ against muscle dysfunction induced by metabolic dysfunction are majorly associated with the regulation of mitochondrial functions. We previously found that TMZ improves exercise capacity by enhancing MQC in healthy C57BL/6 mice^16^. TMZ inhibits fatty acid oxidation rates in the heart, TMZ’s effect on skeletal muscle fatty acid oxidation may be extremely mild and showed a decreased tendency of FFA, but there was no significant difference^49^. We found, that TMZ inhibited the elevation of plasma FA levels and visceral fat accumulation following a HFD, suggesting that TMZ may have effects on the occurrence of metabolic dysfunction. A simultaneous increase in mitochondrial biogenesis, dynamics, and mitophagy in PA-induced muscle cell and HFD-induced obese mice was also observed. TMZ promoted mitochondrial fission to separate dysfunctional mitochondria, which further undergo mitophagy for efficient energy re-utilization. Moreover, TMZ enhances mitochondrial biogenesis to increase mitochondrial population and promotes mitochondrial fusion to maintain MQC.

**Figure 6:**
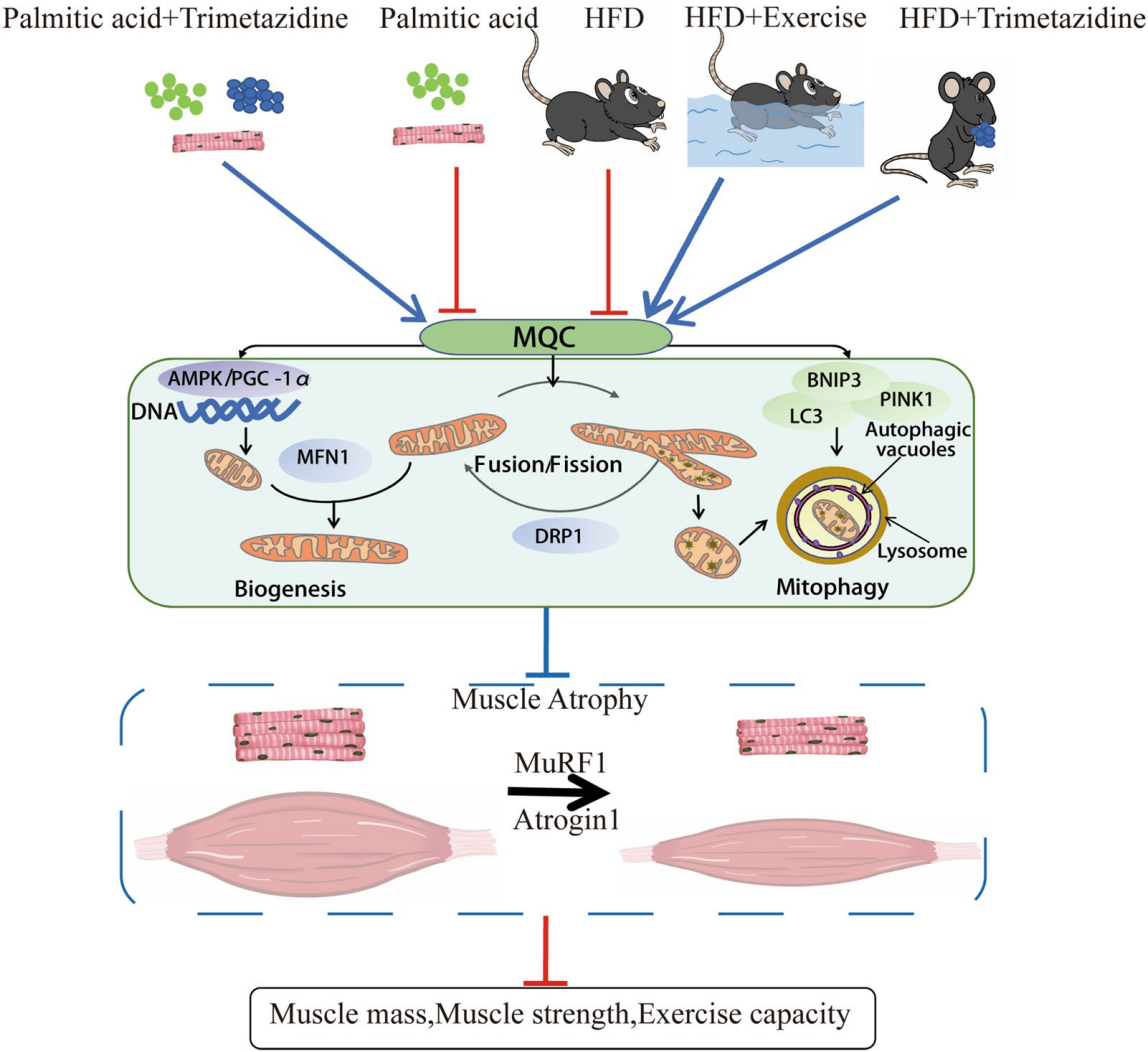
Proposed pathway of TMZ effects on HFD-induced muscle dysfunction. Mitochondrial quality control (MQC) including mitochondrial biogenesis, dynamics, and autophagy cooperate to ensure mitochondrial homeostasis. Mitochondrial dysfunction arising from impaired mitochondrial quality control is critically involved in the pathogenesis of muscle atrophy. During high-fat diet (HFD)-induced obesity, adversely alteration in atrophic factors (i.e. Atrogin-1/MAFbx and MuRF1) and MQC were observed. However, our data shows exercise training or TMZ treatment enhances MQC and suppresses atrophic factors to alleviate HFD-induced muscle dysfunction. Thus, we conclude that TMZ might be a potential therapy in the treatment of muscle atrophy in addition to exercise against HFD-induced muscle dysfunction.

Overall, our data suggests that both exercise and TMZ can improve HFD-induced muscle atrophy and dysfunction. Enhancing skeletal muscle MQC and mitochondrial function may be one of the key mechanisms (Fig. 6). As an anti-angina agent, TMZ has been widely used for the treatment of cardiovascular disease. To date, the present study is the first to show that TMZ could also resist obesity-induced muscle dysfunction comparable to exercise. This suggests, TMZ may be further developed as a potential new therapeutic strategy for patients with obesity, and exercise intolerance, and exercise restriction, such as bed rest and immobilization, hospitalization due to orthopedic surgery, regenerative impairment, or ageing.

## Methods

### Cell culture

C2C12 mouse myoblasts (Cobioer Biotechnology Ltd, Nanjing, China) were cultured in Dulbecco Modified Eagle Medium (DMEM) containing 10% fetal bovine serum and penicillin/streptomycin (5000 U/5000 μg/mL; Gibco, Grand Island, NY, USA). Cells with a 75% confluence were incubated with differentiation media (DMEM containing 2% horse serum, Gibco) for 5 days. Following differentiation, myotubes were incubated with 0.75 mmol/L of PA for 24 h followed with or without 50 μg/mL of TMZ.

### Animal experiments

Sixty-four Male C57BL/6 J mice (8 weeks old) were purchased from the Animal Laboratory Centre of Xiangya Medical School (Changsha, Hunan, China). They were housed in temperature-controlled (22 °C ± 2 °C) quarters with a 12-h shift of light-to-dark cycle and free access to water and food.

After adaptive feeding for one week, mice were randomly divided into four groups (n = 16 per group) including normal diet (ND) group, high-fat diet (HFD) group, HFD + exercise (HFD + EX) group, and HFD + Trimetazidine (HFD + TMZ) group. Fat accounted for 18% and 45% of total calories in the ND and HFD, respectively. The mice in the HFD + EX group underwent an exercise training program (see following parts) while the mice in the HFD + TMZ group were administered with TMZ (10 mg/kg/day, Schweageer Pharmaceuticals, ig). All other groups were given an equal amount of saline. Bodyweight gain was monitored weekly for eight weeks.

**Figure 7:**
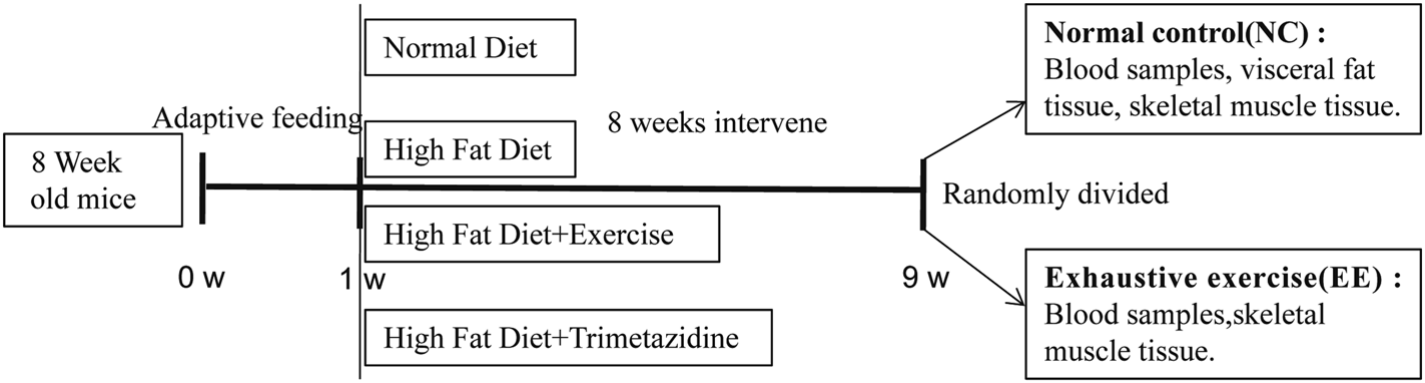
Diagram of animal experiments. After a 1-week adaptive feeding, mice were randomly divided into four groups: normal diet (ND), high-fat diet (HFD), HFD + Trimetazidine (HFD + TMZ), and HFD + exercise (HFD + EX). 8 weeks later, mice in each group were randomly divided into two subgroups: normal control subgroup and exhaustive exercise (EE) subgroup, the subsequent experiments were performed as the diagram shows.

After 8 weeks, each group was further divided into two subgroups with or without exhaustive exercise, respectively (e.g., normal control, NC and exhaustive exercise, EE, n = 8 each group). In the EE subgroup, mice were subjected to an inverted screen test and a loaded swimming (5% of body weight) test as previously described^15^. After the experiments, all mice were anesthetized via an intraperitoneal injection of 1% pentobarbital sodium (150 mg/kg) and then sacrificed via exsanguination. The blood and skeletal muscle samples were eventually obtained from both NC and EE groups. Blood samples were collected from the inferior vena cava. Gastrocnemius muscle and visceral fat including mesenteric, epididymal, and perirenal fat tissue were dissected and weighted. All the procedures involving mice were conducted under the guidelines for the use of live animals of the National Institute of Health and were approved by the Animal Ethics Committee of Xiangya Medical School, Central South University (Changsha, China) (approval ID: SYXK 2015-0017), and in accordance with the ethical standards laid down in the 1964 Declaration of Helsinki and its later amendments. Furthermore, this study was conducted in accordance with the ARRIVE guidelines and care was taken to avoid any unnecessary animal suffering. The protocol for the animal experiments has been described in Fig. 7.

### Exercise training and exhaustive exercise

The HFD + EX group was subjected to moderate intensity swim training as previously described^15,16^. Briefly, the mice were placed in a Morris water maze pool (XR-XM101-R, ZSdichuang, Beijing, China, with a diameter of 120 cm and a depth of 60 cm) and swam 10 min on the first day. The swimming time was increased 10 more minutes daily until reaching 60 min/day. Following swimming, the mice were then dried gently with towels and blowers. The swimming test was conducted between 9 a.m. and 2 p.m. when mice exhibited minimal variations in aerobic capacity.

The EE subgroups were also subjected to a forced weight-loaded swimming test with a lead sheath (0.8 mm thick, 0.5 cm wide) weighed at 5% of their body weight. The mice were forced to swim till “exhaustion”, defined as the duration of forced weight-loaded swimming until the mouth and nose of the mouse were completely submerged in water for up to 7 s, and the exhaustive swimming time was recorded and analyzed^50^. The condition and equipment used in this program were the same as those used in the exercise training protocol.

### Inverted screen test and exercise capacity

The limb strength was evaluated by an inverted screen test. Briefly, mice were placed in the center of an invertible 40 × 40 cm wire screen and mounted 80 cm above a padded surface. After gently inverting the screen, the time of hanging and limb strength was recorded. Exercise capacity was assessed by the time of the forced weight-loaded swimming test mentioned above in the EE session.

### Western blot analysis

Muscle tissues or cells were lysed and proteins were extracted for immunoblotting with antibodies including P-AMPK (5256S, CST), CS (16131-1-AP, Proteintech), LC3 (14600-1-AP, Proteintech), PINK1 (23274-1-AP, Proteintech), BNIP3 (ab109362, abcam), PGC-1α (66369-1-Ig, Proteintech), DRP1 (12957-1-AP, Proteintech), MFN1 (13798-1-AP, Proteintech), Atrogin1 (12866-1-AP, Proteintech), MuRF1 (55456-1-AP, Proteintech), MyHC-II (66212-1-Ig, Proteintech), and Glyceraldehyde 3-phosphate dehydrogenase (GAPDH) (10494-1-AP, Proteintech, Rosemont, IL, USA).

### Gene expression assay

Total DNA was extracted from skeletal (quadriceps femoris) muscles by TRIzol (Invitrogen, CA, USA), using a DNeasy Kit (Qiagen). MtCO3 oligos and succinate dehydrogenase complex subunit A (SDHA) were analyzed to evaluate the quantification of mitochondrial and nuclear genomes. Expressions of Mitochondrial DNA (mtDNA) were quantified by real-time reverse transcription PCR (RT-qPCR) analysis by using the following primers, as shown in Table 1.

**Table 1:** Sequence of primers used for RT-qPCR assays.

Gene	Primer	Product length (bp)
M-DNA-mt-Co3	FGCAGGATTCTTCTGAGCGTTCT	67
	RGTCAGCAGCCTCCTAGATCATGT	
M-DNA-Sdha	FTACTACAGCCCCAAGTCT	194
	RTGGACCCATCTTCTATGC	
GAPDH	FGCGACTTCAACAGCAACTCCC	122
	RCACCCTGTTGCTGTAGCCGTA	

### MQC assessment

For the assessment of mitochondrial quality control (MQC), the expression levels of mitophagy (LC3, PINK1, BNIP3), mitochondrial dynamics (DRP1, MFN1), and biogenesis (P-AMPK, PGC-1α) were determined by Western blot. LysoTracker and mitoTacker (Yeasen) were used to label mitolysosomes which were observed by a laser scanning confocal microscope (LSCM, Olympus, USA).

### ATP content

The ATP content in muscle tissues was determined via the phosphomolybdic acid colorimetric method, according to the manufacturer’s instructions (A095-1-1, Nanjing Jiancheng Bioengineering Institute, Nanjing, China).

### Measurement of cell OCR

C2C12 myoblasts were seeded in XF 24-well microplates (Seahorse Bioscience, Billerica, USA) and differentiated. Mitochondrial inhibitors, including 1 μmol/L oligomycin, 1 μmol/L carbonyl cyanide 4-(trifluoromethoxy) phenylhydrazone (FCCP), and 1 μmol/L rotenone/antimycin A, were incubated with these cells respectively. The cell OCR was measured by the extracellular flux analysis (Seahorse Biosciences) every 8 min.

Basal respiration represents the baseline oxygen consumption reading prior to the addition of inhibitors, antimycin A Rotenone, with the subtraction of non-mitochondrial respiration. Maximal respiration is shown as the maximum OCR measurement value following FCCP injection and with the subtraction of non-mitochondrial respiration. ATP production respiration represents the OCR reduction after the addition of an inhibitor of ATP synthase, oligomycin. Spare respiratory capacity is calculated by maximal respiration subtracting basal respiration. After detection, cell protein content was calculated, and OCR was adjusted accordingly.

### Immunofluorescence staining

The cells were fixed with 4% paraformaldehyde and stained with anti-MyHC-II antibody for 1 h at 37 ℃. Nuclei were stained with Hoechst 33342. The images were acquired using a fluorescence microscope (Eclipse, Nikon, Japan).

### Transmission electron microscope

Myotubes were fixed with 2.5% glutaraldehyde in 0.1 M phosphate buffer and observed under a transmission electron microscope (TEM) (Tecnai G2 Spirit, FEI, USA) and then fixed by 2.5% Glutaraldehyde and 1% osmic acid. They were then washed with 0.1 mol/L phosphate buffer, the tissue was dehydrated with acetone at a gradient concentration. The tissue was embedded and solidified in 37 ℃ for 12 h and 60 ℃ for 24 h, then sliced to 50–100 nm, and eventually, they were examined via TEM.

### Biochemical analyses

Blood samples were obtained after 12-h fasting from NC subgroups and then assayed by a serum-free fatty acid assay kit (A042, Nanjing Jiancheng Bioengineering Institute, China). Blood samples were obtained after EE and fasting in EE subgroups, and then assayed by serum CK and BUN by assay kits, respectively (A032, C013-2, Nanjing Jiancheng Bioengineering Institute).

### HE staining

Gastrocnemius muscle tissues were fixed overnight with 4% paraformaldehyde and stained with hematoxylin–eosin (HE). The stained tissues were subsequently observed via an Olympus microscope. The image analysis software Image J was used to measure the area of each muscle fiber. Eventually, we statistically analyzed the difference among groups.

### Statistical analysis

All the results are expressed as the Mean ± standard error of the mean (SEM). One-way ANOVA with Newman–Keul’s tests was used to determine differences between group mean values. The statistical significance was set at p < 0.05. Statistical analysis was performed using Prism 6 software (GraphPad, Inc.).

### Ethics statement

All the procedures involving mice were conducted under the guidelines for the use of live animals of the National Institute of Health and were approved by the Animal Ethics Committee of Xiangya Medical School, Central South University (Changsha, China) (approval ID: SYXK 2015-0017), and in accordance with the ethical standards laid down in the 1964 Declaration of Helsinki and its later amendments. Furthermore, this study was conducted in accordance with the ARRIVE guidelines.

## Supplementary Information

Supplementary Information.

## Data Availability

The original contributions presented in the study are included in the article/“Supplementary Material”, further inquiries can be directed to the corresponding authors Y.D. and S.L.
