# Supplementary material for: Trimetazidine and exercise provide comparable improvements to high fat diet-induced muscle dysfunction through enhancement of mitochondrial quality control
Source: Sci Rep. 2021 Sep 27;11:19116. doi: 10.1038/s41598-021-98771-6 (PMC8476493; doi:10.1038/s41598-021-98771-6)
Supplement: Supplementary file 1 — Supplementary Information. [file 41598_2021_98771_MOESM1_ESM.pdf]

Supplementary Appendix

Trimetazidine and Exercise Provide Comparable Improvements to High Fat Diet-induced Muscle Dysfunction through Enhancement of Mitochondrial Quality Control

Wenliang Zhang[1], Baiyang You[1], Yaoshan Dun[1,2], Dake Qi[3], Ling Qiu[1], Jeffrey W. Ripley-Gonzalez[1], Fan Zheng[1], Siqian Fu[1], Cui Li[1] and Suixin Liu[1,4]*

1. Division of Cardiac Rehabilitation, Department of Physical Medicine & Rehabilitation, Xiangya Hospital of Central South University, Changsha, Hunan, China.
2. Division of Preventive Cardiology, Department of Cardiovascular Medicine, Mayo Clinic, Rochester, MN, USA.
3.College of Pharmacy, University of Manitoba, Winnipeg, Canada
4. National Clinical Research Center for Geriatric Disorders, Xiangya Hospital of Central South University, Changsha, Hunan, China.

* Corresponding Author. Suixin Liu, MD, Ph.D., Division of Cardiac Rehabilitation, Department of Physical Medicine & Rehabilitation, Xiangya Hospital of Central South University, 87 Xiangya Road, Changsha, Hunan, 410008, P.R. China. E-mail, liusuixin@csu.edu.cn (SXL); +86 (731)84327174

This supplementary appendix was created by the authors to provide all original gels/blots images for review.

Supplementary Fig.1 is the original gel/blots shown in figure 1 of the main manuscript, Supplementary Fig.2 is the original gel/blots shown in figure 2 of the main manuscript and so on.

In **Fig. 3.** TMZ promotes PA-attenuated mitochondrial quality control (MQC) and functions in muscle cells. The original data presents P-AMPK, PGC-1α, LC3II/LC3I, BNIP3, DRP1, MFN1 and CS protein expressions were determined in four groups, we just cropped the right most group in the data analysis. In **Fig. 5.** TMZ regulates MQC and improves mitochondrial functions in HFD mice. The original data presents PGC-1α, LC3II/LC3I, PINK1, DRP1 and MFN1 and CS protein expressions were determined in five groups, we just cropped the right most group in the data analysis.

Supplementary Fig. 1. TMZ prevents palmitate (PA)-induced muscle cell damage

Atrogin1	GAPDH
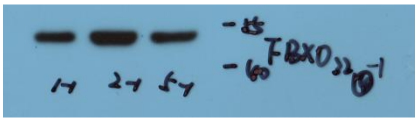	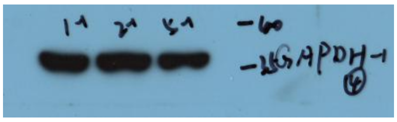
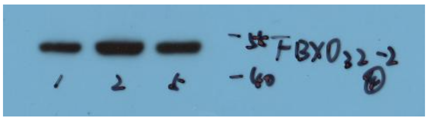	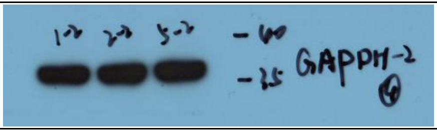
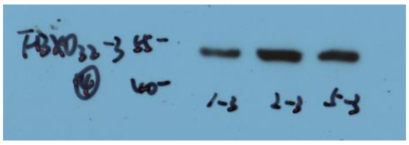	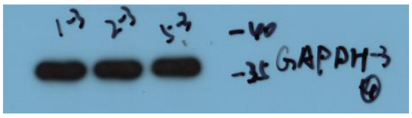

MuRF1	GAPDH
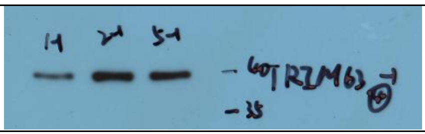	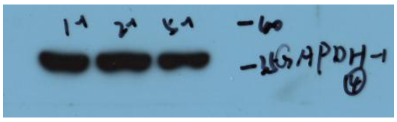
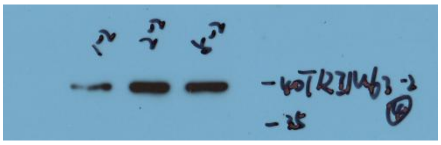	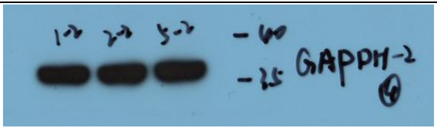
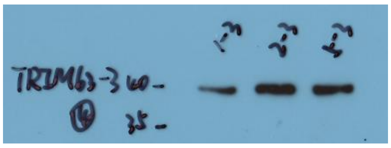	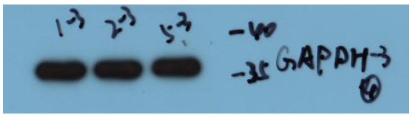

MyHC-II	GAPDH
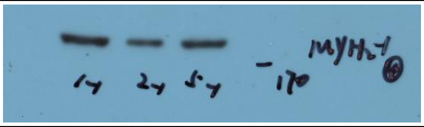	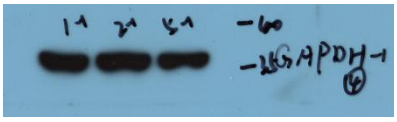
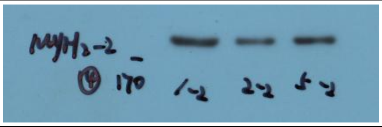	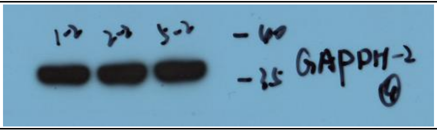
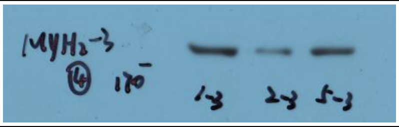	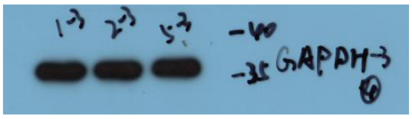

Supplementary Fig. 2. TMZ promotes PA-attenuated mitochondrial quality control (MQC) and functions in muscle cells

P-AMPK	GAPDH
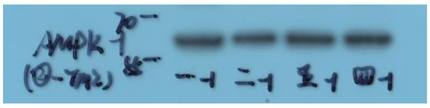	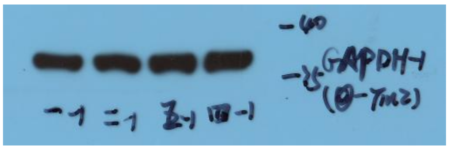
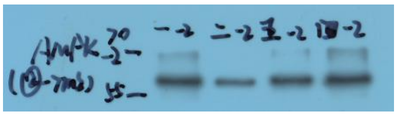	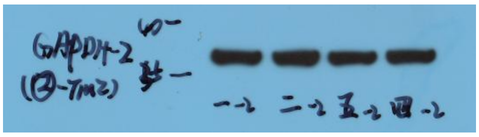
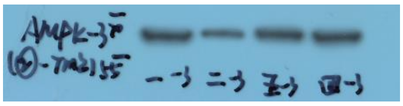	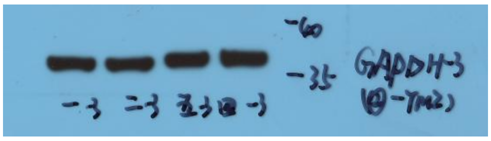

PGC-1α	GAPHD
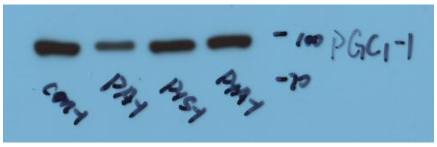	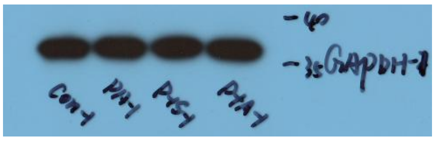

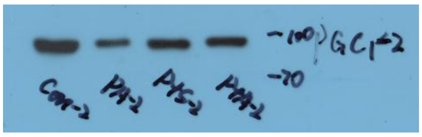	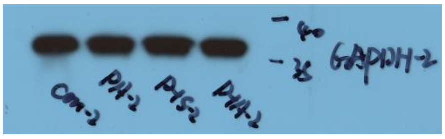
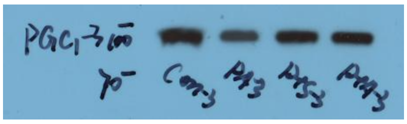	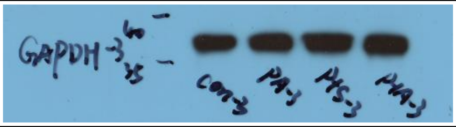

LC3II/LC3I	GAPDH
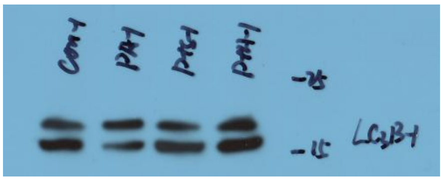	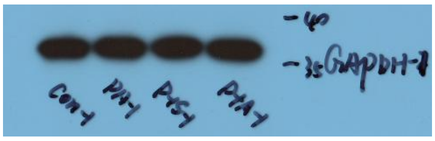
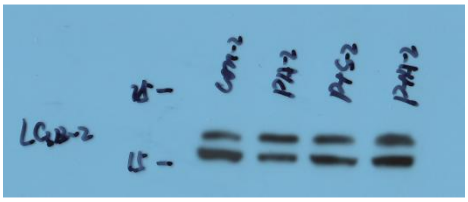	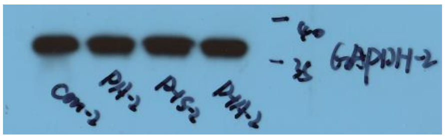
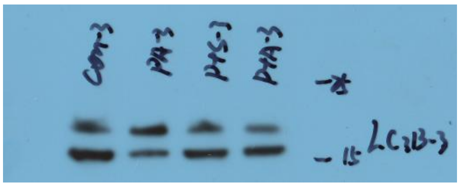	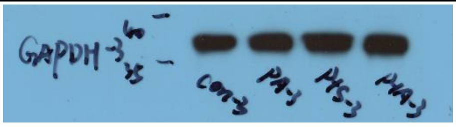

BNIP3	GAPDH
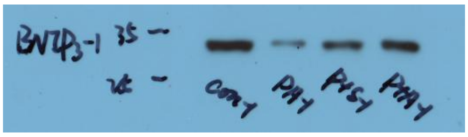	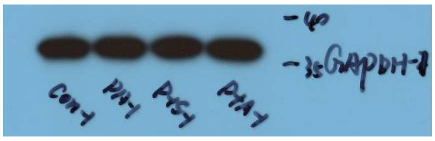
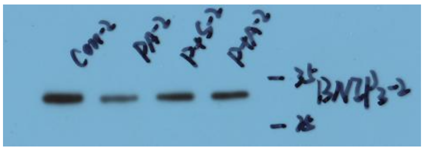	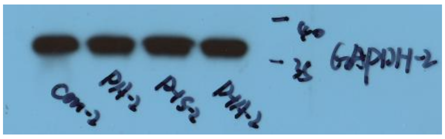
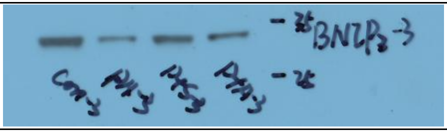	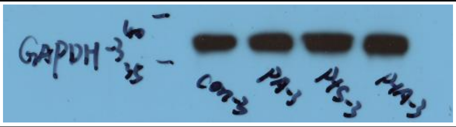

DRP1	GAPDH
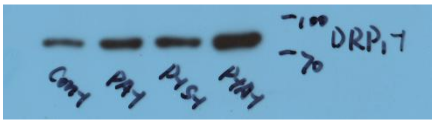	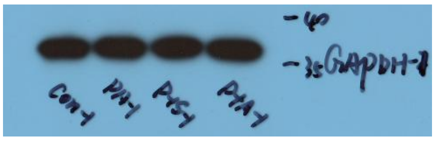
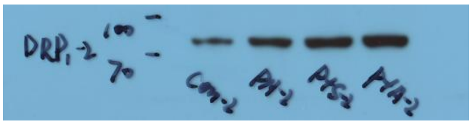	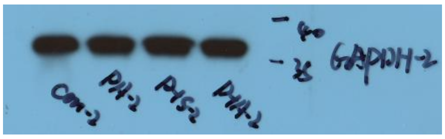
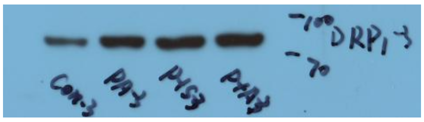	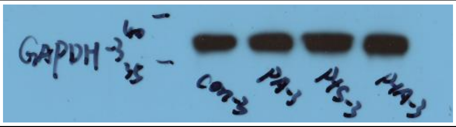

MFN1:	GAPDH
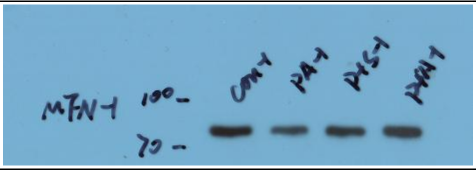	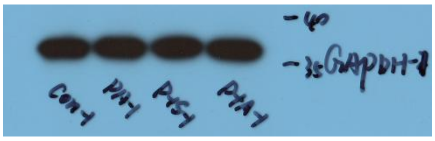
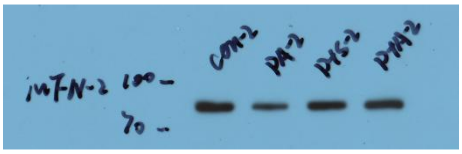	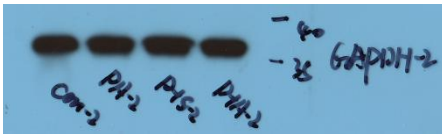
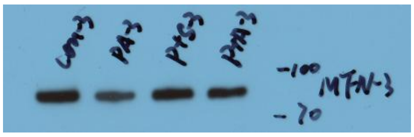	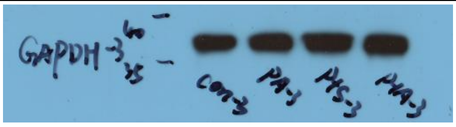

CS	GAPDH
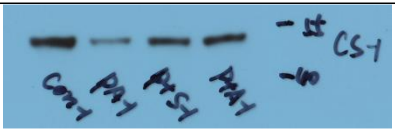	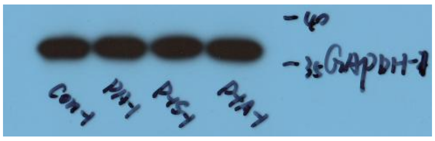

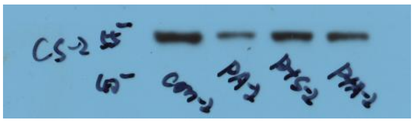	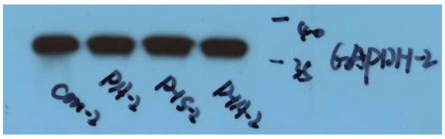
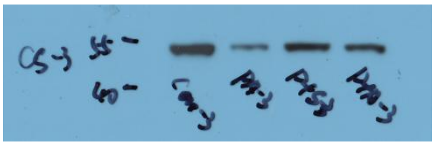	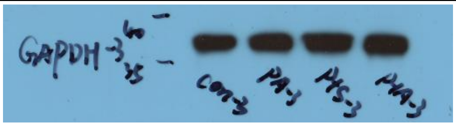

Supplementary Fig. 3. TMZ prevents metabolic dysfunction and muscle atrophy in mice following high-fat diet feeding as exercise

Atrogin1	GAPDH
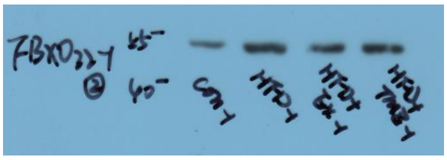	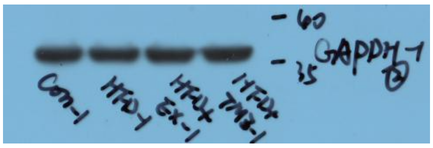
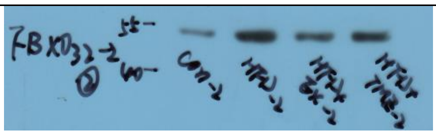	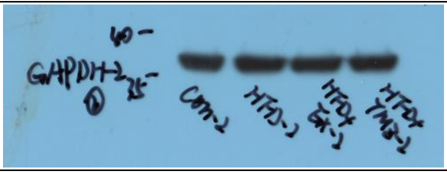
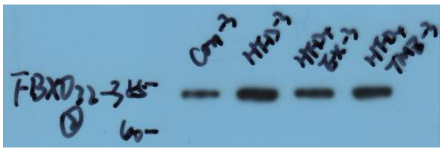	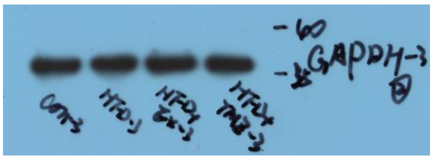

MuRF1	GAPDH
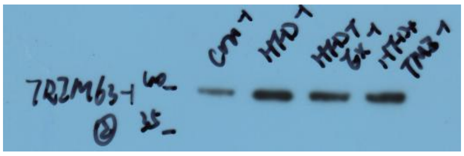	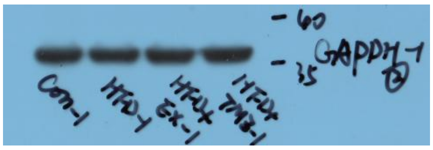
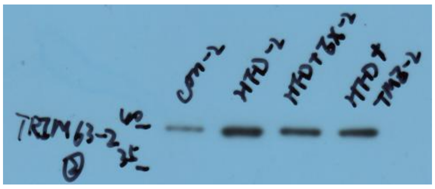	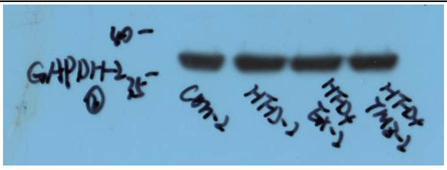

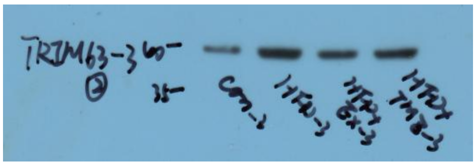	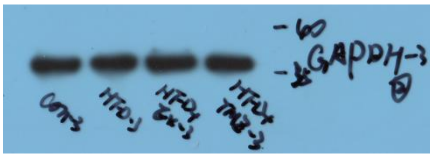

MyHC-II	GAPDH
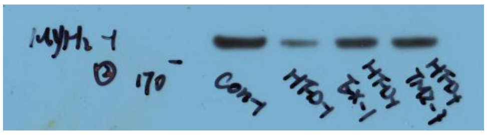	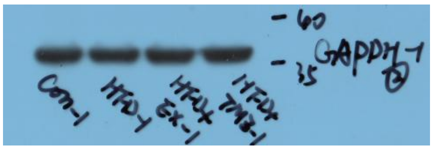
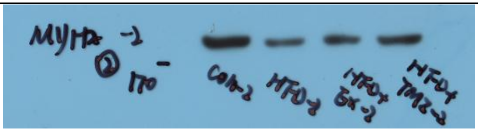	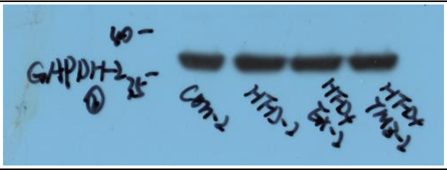
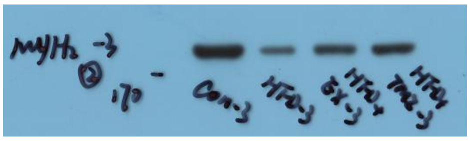	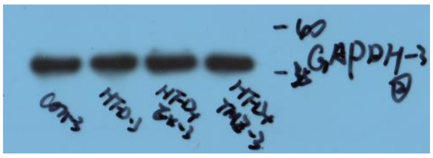

Supplementary Fig. 4. As exercise, TMZ regulates MQC and improves mitochondrial functions in HFD mice

P-AMPK,:	GAPDH
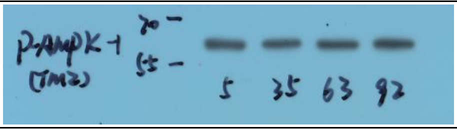	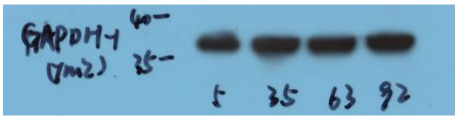
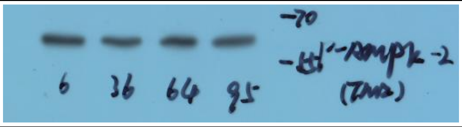	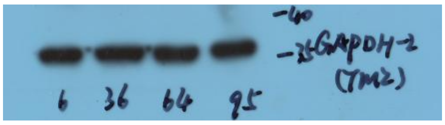
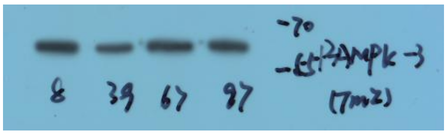	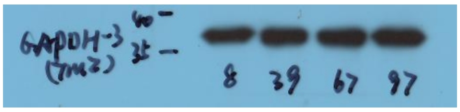

PGC-1α,:	GAPDH

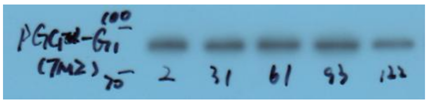	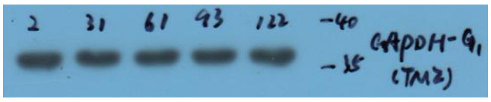
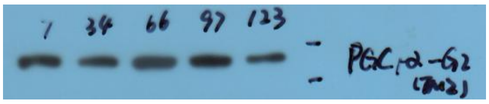	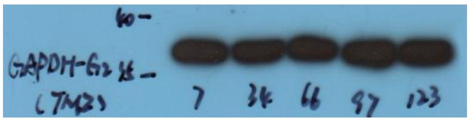
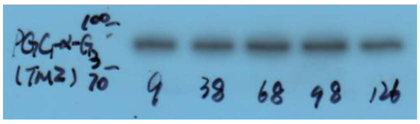	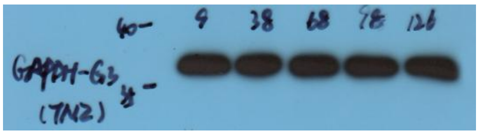

LC3II/LC3I,:	GAPDH
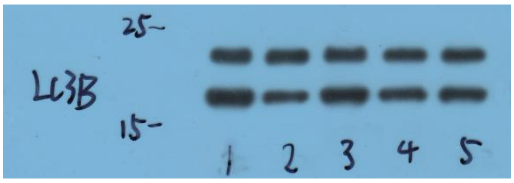	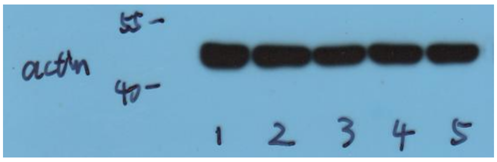
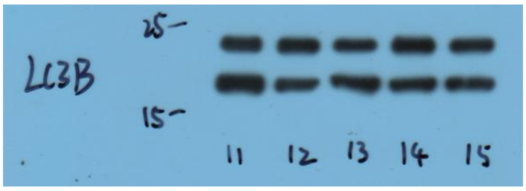	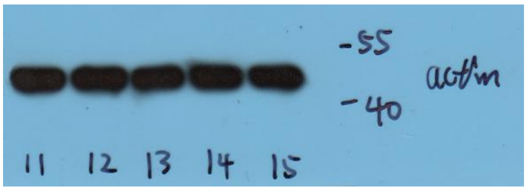
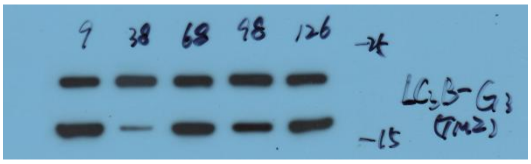	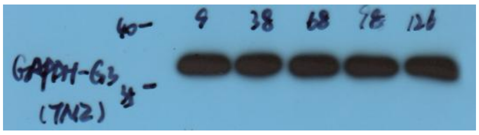

PINK1	ACTIN
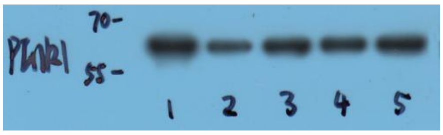	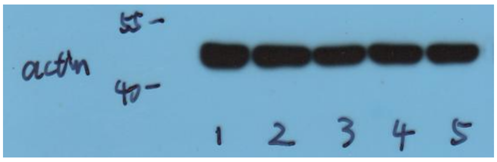
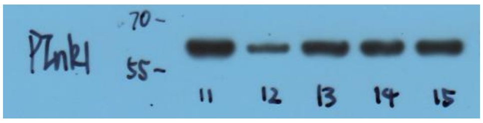	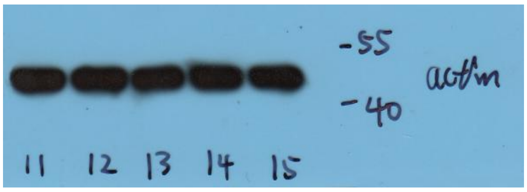

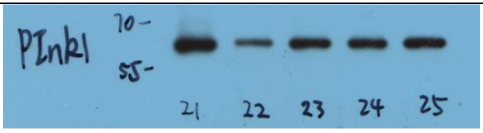	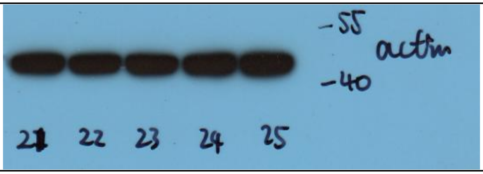

DRP1	GAPDH
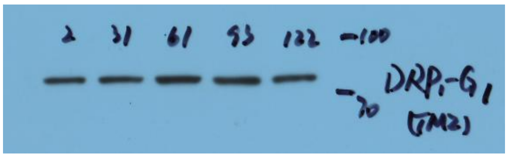	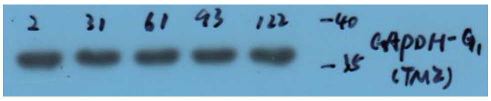
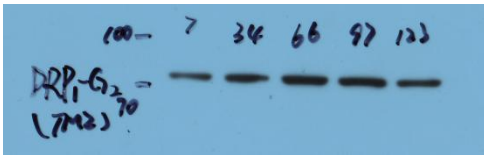	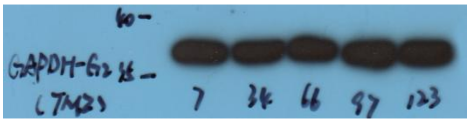
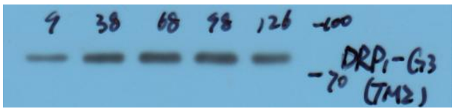	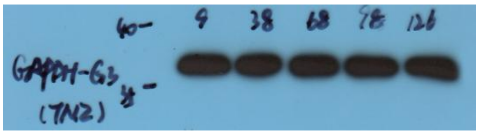

MFN1:	GAPDH
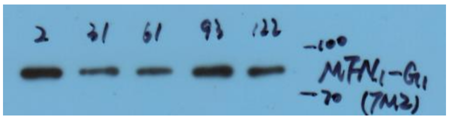	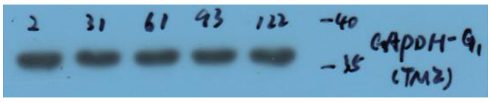
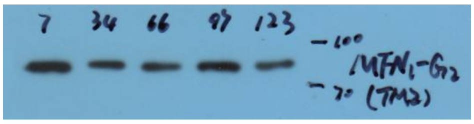	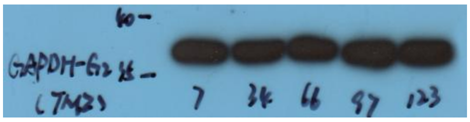
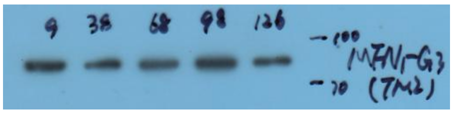	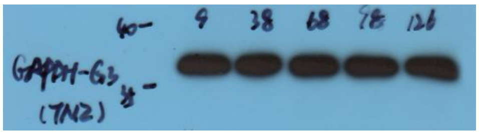

CS	GAPDH
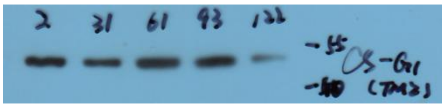	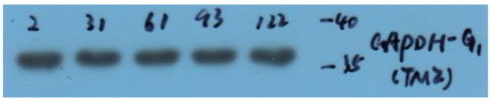

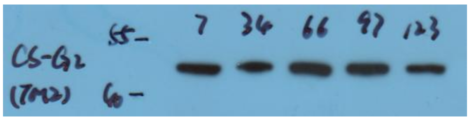
CS-G2
(TMZ)
55
60
7 34 66 97 123

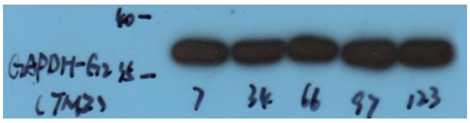
60
GAPDH-G2
(TMZ)
7 34 66 97 123

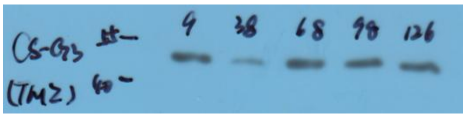
CS-G3
(TMZ)
9 38 68 98 126

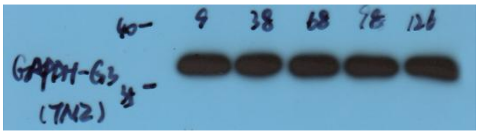
60
GAPDH-G3
(TMZ)
9 38 68 98 126
